# Effect of *Toona microcarpa* Harms Leaf Extract on the Coagulation System

**DOI:** 10.1155/2014/615363

**Published:** 2014-04-10

**Authors:** Hao Chen, Min Jin, Yi-Fen Wang, Yong-Qing Wang, Ling Meng, Rong Li, Jia-Ping Wang, Li Gao, Yi Kong, Ji-Fu Wei

**Affiliations:** ^1^School of Life Science & Technology, China Pharmaceutical University, Nanjing 210009, China; ^2^Research Division of Clinical Pharmacology, The First Affiliated Hospital, Nanjing Medical University, Nanjing 210029, China; ^3^State Key Laboratory of Phytochemistry and Plant Resources in West China, Kunming Institute of Botany, Chinese Academy of Sciences, Lanhei Road, Heilongtan, Kunming 650201, China

## Abstract

*Toona microcarpa* Harms is a tonic, antiperiodic, antirheumatic, and antithrombotic agent in China and India and an astringent and tonic for treating diarrhea, dysentery, and other intestinal infections in Indonesia. In this study, we prepared ethyl-acetate extract from the air-dried leaves of * Toona microcarpa* Harms and investigated the anticoagulant activities * in vitro* by performing activated partial thromboplastin time (APTT), prothrombin time (PT), and thrombin time (TT) assays. Antiplatelet aggregation activity of the extract was examined using adenosine diphosphate (ADP), collagen, and thrombin as agonists, and the inhibitions of factor Xa and thrombin were also investigated. Bleeding and clotting times in mice were used to determine its anticoagulant activities * in vivo*. It is found that * Toona microcarpa * Harms leaf extract (TMHE) prolonged APTT, PT, and TT clotting times in a dose-dependent manner and significantly inhibited platelet aggregation induced by thrombin, but not ADP or collagen. Clotting time and bleeding time assays showed that TMHE significantly prolonged clotting and bleeding times * in vivo*. In addition, at the concentration of 1 mg/mL, TMHE inhibited human thrombin activity by 73.98 ± 2.78%. This is the first report to demonstrate that THME exhibits potent anticoagulant effects, possibly via inhibition of thrombin activity.

## 1. Introduction

Many traditional Chinese herbal medicines have been used for thousands of years in clinical practice because of their proven efficacy, wide indications, high safety profile, and low toxicity [1].* Toona microcarpa* Harms, a tree reaching 10 m, is a perennial hardwood of the family* Meliaceae* that is found in India, Bhutan, Laos, Malaysia, Myanmar, Papua New Guinea, Thailand, Sikkim, Indochina, and southern China. This species yields excellent timber and has long been used as a traditional Chinese medicine (TCM) for treating various conditions as its leaves, seeds, and root bark have medicinal effects. Specifically, the bark is used as a powerful astringent and purgative, and the leaf extract has antithrombotic effect and antibiotic activity against* Staphylococcus*, with leaf tip concoctions applied to swellings.* Toona microcarpa* Harms is considered a tonic, antiperiodic, antirheumatic, and antithrombotic agent in China and India and is used as an astringent and tonic for treating diarrhea, dysentery, and other intestinal infections in Indonesia [2]. However, there are few reports about the antithrombotic activities of* Toona microcarpa* Harms and the mechanism is unknown.

Thrombosis is a major cause of morbidity and mortality and is closely related to activated platelet adhesion, aggregation, secretion functions, and activation of intrinsic and extrinsic coagulation systems, which cause blood coagulation and fibrin formation [3]. In TCM, thrombotic disorders are described as blood stasis syndrome.* Toona microcarpa* Harms has some effects on activating blood circulation to dissipate blood stasis; however, the mechanism underlying its effect has been poorly studied. In this study, we prepared ethylacetate extract from the air-dried leaves of* Toona microcarpa* Harms and investigated its antithrombotic activity and underlying mechanism.

## 2. Materials and Methods

### 2.1. Preparation of* Toona microcarpa* Harms Leaf Extract (TMHE)

The aerial parts of* Toona microcarpa* Harms were collected in February 2013 from the Jinghong region of Yunnan Province, China. The plant was identified by Dr. Rong Li, and a voucher specimen (KIB 13-02-08) was deposited in the State Key Laboratory of Phytochemistry and Plant Resources in west China, Kunming Institute of Botany, Chinese Academy of Sciences. Air-dried and powdered leaves of* Toona microcarpa* Harms (1 kg) were extracted with 90% ethanol (5000 mL × 2) at room temperature and concentrated* in vacuo* to yield crude extract. The dry extract was resuspended in distilled water (1000 mL) and extracted twice with petroleum (30–60°C) to remove pigments and lipids, followed by two more extractions with ethylacetate using liquid-liquid partitioning. After removing the solvent using a rotary vacuum evaporator, the ethylacetate fraction was used to determine its bioactivity. TMHE was dissolved in dimethylsulfoxyde to obtain stock solutions of 50 mg/mL. Working solutions were obtained by dilution with distilled water.

### 2.2. Activated Partial Thromboplastin Time (APTT), Prothrombin Time (PT), and Thrombin Time (TT) Assays* In Vitro*

Male imprinting control region (ICR) mice (28–32 g) were supplied by the animal center of Nanjing Medical University. All animal experiments were approved by the Animal Care and Use Committee of The First Affiliated Hospital of Nanjing Medical University. Blood was drawn from the eyeball of ICR mice and separately centrifuged at 3000 rpm for 15 min to obtain platelet poor plasma (PPP). For* in vitro *APTT assays, 50 *μ*L normal citrated PPP was incubated with 50 *μ*L TMHE (0.5, 1, 2, 3, or 4 mg/mL) and 50 *μ*L APTT reagent for 3 min at 37°C. APTT clotting time was immediately recorded after the addition of 100 *μ*L calcium chloride (20 mM). For* in vitro* PT assays, 50 *μ*L normal citrated PPP was incubated with 50 *μ*L (0.5, 1, 2, 3, or 4 mg/mL) TMHE for 3 min at 37°C. Clotting time was immediately recorded after the addition of 100 *μ*L PT reagent [4]. For* in vitro* TT assay, 100 *μ*L normal citrated PPP was incubated with 100 *μ*L (0.5, 1, 2, 3, or 4 mg/mL) TMHE for 2 min at 37°C. Clotting time was immediately recorded after the addition of 100 *μ*L TT reagent [5]. All coagulation assays were performed in triplicate. Heparin (1 mg/mL) and argatroban (TIPR Pharmaceutical Responsible Co., Ltd., 0.05 mg/mL) were used as positive controls, and the extract solvents were used as negative controls.

### 2.3. Platelet Aggregation Test

Male New Zealand rabbits (4-5 kg) were supplied by the animal center of Nanjing Medical University. All animal experiments were approved by the Animal Care and Use Committee of The First Affiliated Hospital of Nanjing Medical University. After application of the local anesthetic lidocaine, blood was drawn from the carotid artery of male New Zealand rabbits and directly collected into vials containing sodium citrate (1 : 9 v/v) mixture. The blood samples were centrifuged at 1000 rpm for 10 min at room temperature to prepare platelet rich plasma (PRP), and the residue was centrifuged at 3000 rpm for 15 min at room temperature to obtain PPP [6]. Briefly, 270 *μ*L PRP and 30 *μ*L TMHE were incubated at 37°C in an aggregometer. After a 3 min preincubation, 30 *μ*L of agonists (ADP, collagen, or thrombin, the final concentrations are 5 *μ*M, 2 *μ*L/mL, and 1 NIH/mL) was added to initiate aggregation, which was monitored for 6 min. The extract solvents were used as negative controls; aspirin (1 mg/mL) and argatroban (0.005 mg/mL) were used as positive controls. The inhibition rate was calculated as follows: inhibition rate = (Av – At)/Av × 100%; Av is the platelet aggregation percent of negative control and At is the platelet aggregation percent of the TMHE group, respectively.

### 2.4. Thrombin Inhibition Assay

TMHE (20 *μ*L) (0.1, 0.2, 0.3, 0.5, 0.8, or 1 mg/mL) and 20 *μ*L human thrombin (5 NIH/mL) (Hyphen-BioMed, France) in 20 *μ*L Tris-HCl buffer (0.05 M, pH 7.5) were incubated for 15 min in a 96-well plate. The reaction was initiated by adding 20 *μ*L thrombin chromogenic substrate CS-01(38) (2.5 mg/mL), and the absorbance at 405 nm was recorded every 0.5 min for 5 min. The background absorbance was measured just before adding the substrate. In A-t curve, the curve slope was considered the reaction rate (*v*). The enzyme inhibition percentage (*I*) was determined as follows: *I* = (*V*_0_ − *V*_*i*_)/*V*_0_ × 100%. *V*_0_ is the rate of extract solvents, and *V*_*i*_ is the rate of TMHE. Extract solvents were used as negative controls, whereas argatroban (TIPR Pharmaceutical Responsible Co., Ltd., 5 *μ*g/mL) was used as a positive control. The results were expressed as mean ± SD for three independent experiments.

### 2.5. Factor Xa Inhibition Assay

The same protocol as described for thrombin was followed using 20 *μ*L human factor Xa (2.5 *μ*g/mL) (Hyphen-BioMed) in 20 *μ*L PBS buffer (1/15 M, pH 8.34) and 20 *μ*L CS-11(22) substrate (2.5 mg/mL). Rivaroxaban (Bayer, 0.5 *μ*g/mL) was used as a positive control. The results were expressed as mean ± SD for three independent experiments.

### 2.6. APTT and PT Assays* Ex Vivo*

ICR mice (18–22 g) were divided into five treatment groups (both sexes, six per group) and orally administered extract solvents (control), low dose TMHE (20 mg/kg body weight), medium dose TMHE (40 mg/kg body weight), high dose TMHE (80 mg/kg body weight), and dabigatran etexilate (Boehringer Ingelheim, 20 mg/kg body weight). Blood was collected intracardially at 120 min after dosing [5]. The APTT and PT assays were performed as described in Section 2.2, except that test samples were not added to the blood samples.

### 2.7. Clotting Time Assay* In Vivo*

Whole blood clotting time in mice was measured by the capillary glass tube method [7]. ICR mice (18–22 g) were divided into five groups (both sexes, six per group) and orally administered extract solvents (control), low dose TMHE (20 mg/kg body weight), medium dose TMHE (40 mg/kg body weight), high dose TMHE (80 mg/kg body weight), and dabigatran etexilate (20 mg/kg body weight). Each group was administered drug for four consecutive days. Ninety minutes after the last administration, blood samples were collected via the retroorbital plexus with a glass capillary tube and kept on a slide to allow clotting. The blood was stirred with a dry needle every 30 s until the needle wire provoked a fibrous protein, which was defined as clotting time [3].

### 2.8. Bleeding Time Assay* In Vivo*

ICR mice (18–22 g) were divided into five groups (both sexes, six per group) and orally administered extract solvents (control), low dose TMHE (20 mg/kg body weight), medium dose TMHE (40 mg/kg body weight), high dose TMHE (80 mg/kg body weight), and dabigatran etexilate (20 mg/kg body weight). Each group was administered drug for four consecutive days. Ninety minutes after the last administration, the mice tails were marked with a tag approximately 5 mm long and then cut at the mark. Then, the tip of the tail was immersed in saline at 37°C, and the time from cutting the tip of the tail to stopping the bleeding was recorded; this interval was defined as bleeding time [8].

### 2.9. Acute Toxicity

The acute toxicity of TMHE was evaluated in mice according to the description of Wang et al. [9]. Six ICR mice (18–22 g) of both sexes were orally administered with TMHE (2 g/kg body weight) by gavage. Four hours after administration, the mice were observed for toxic symptoms continuously. Finally, the number of survivors was noted after 24 h and these animals were then maintained for further 13 days with observations made daily.

## 3. Results

### 3.1. APTT, PT, and TT Assays* In Vitro*

**Figure 1:**
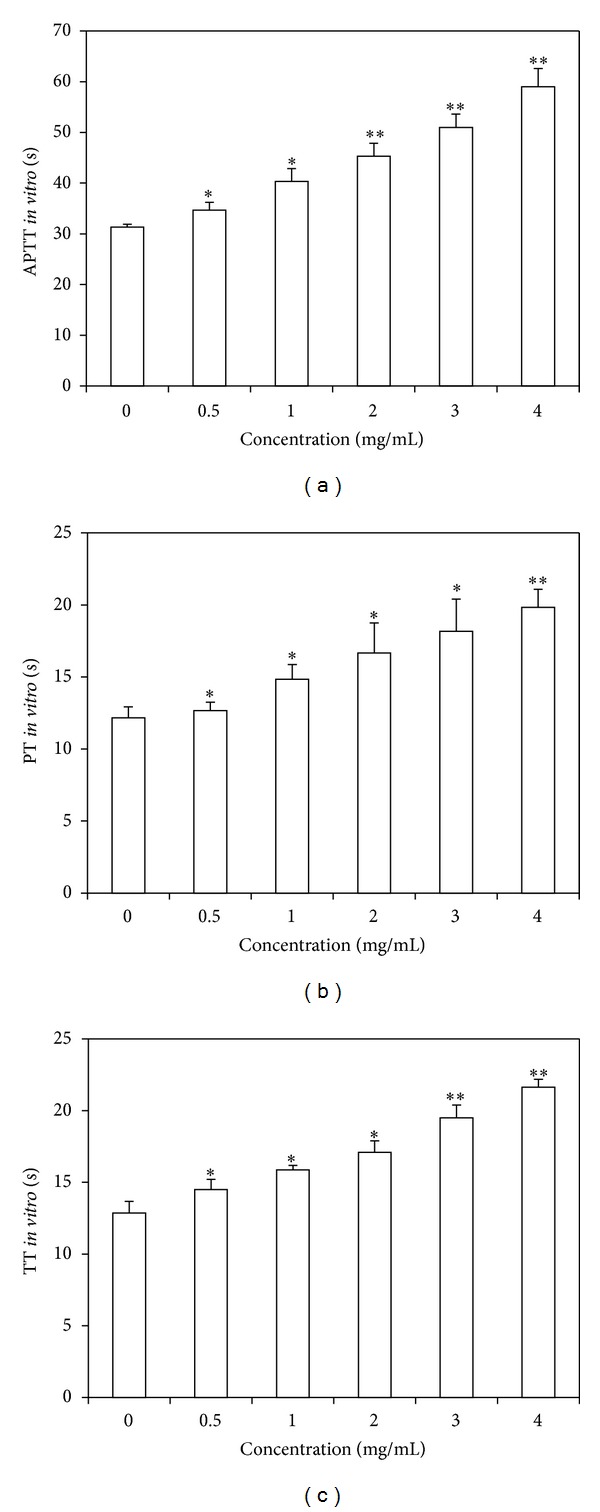
Anticoagulant assays* in vitro*. (a) TMHE prolonged the APTT clotting time in a dose-dependent manner. (b) TMHE prolonged PT clotting time in a dose-dependent manner. (c) TMHE prolonged PT clotting time in a dose-dependent manner. **P < 0.01, *P < 0.05, compared with extract solvent.

For* in vitro* coagulation assays, TMHE prolonged APTT, TT, and PT clotting times in a dose-dependent manner (Figure 1). It prolonged APTT clotting time from 34.67 ± 1.53 to 59 ± 3.61 s (Figure 1(a)), PT clotting time from 12.67 ± 0.76 to 19.83 ± 1.26 s (Figure 1(b)), and TT clotting time from 14.5 ± 0.7 to 21.63 ± 0.55 s (Figure 1(c)) at the concentration of 4 mg/mL. Heparin prolonged APTT and PT clotting times more than 120 s and 60 s, respectively, at a concentration of 1 mg/mL. Argatroban prolonged TT clotting times more than 120 s at a concentration of 0.05 mg/mL.

### 3.2. *In Vitro* Antiplatelet Aggregation Assay

The potential antiplatelet aggregation activity of TMHE was investigated using antiplatelet aggregation assays using ADP or collagen or thrombin as agonists. TMHE at a concentration up to 8 mg/mL did not significantly inhibit platelet aggregation induced by the two platelet agonists. However, TMHE inhibited the thrombin stimulated platelet aggregation activity by 31.41 ± 7.84% at the concentration of 1 mg/mL. Argatroban inhibited the thrombin stimulated platelet aggregation activity by 78.21 ± 3.29% at the concentration of 0.005 mg/mL.

### 3.3. Thrombin and Factor Xa Inhibition Assays

**Figure 2:**
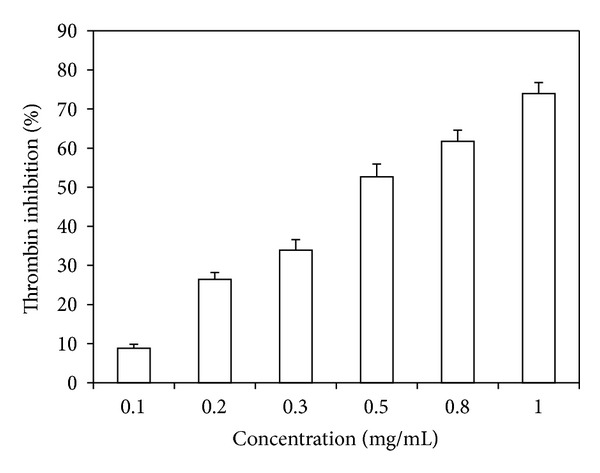
Thrombin inhibition of TMHE. TMHE inhibited the activity of thrombin in a dose-dependent manner.

As shown in Figure 2, TMHE inhibited the activity of human thrombin in a dose-dependent manner. Specifically, it inhibited human thrombin activity by 73.98 ± 2.78% at a concentration of 1 mg/mL, whereas at a concentration of 5 *μ*g/mL argatroban inhibited thrombin activity by 36.32 ± 2.24%. However, TMHE did not inhibit the activity of human factor Xa.

### 3.4. APTT and PT Assays* Ex Vivo*

**Figure 3:**
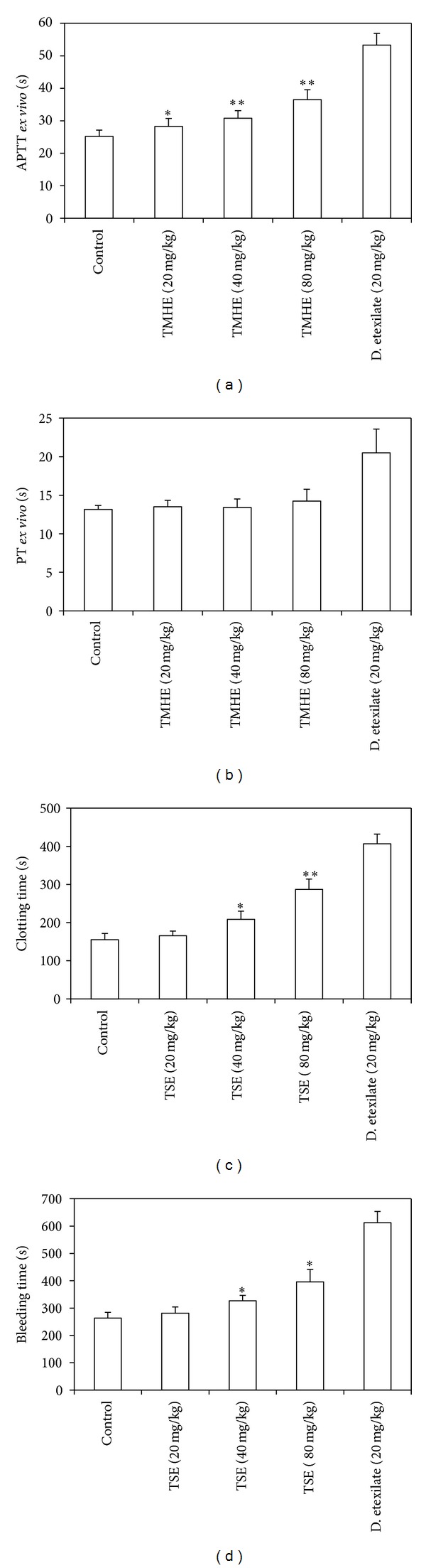
Anticoagulant assays* in vivo*. (a) APTT clotting time increased at medium and high doses of TMHE. (b) There were no significant changes in PT clot time. (c) Effect of TMHE on clotting time. (d) Effect of TMHE on bleeding time. **P < 0.01, *P < 0.05, compared with control group.

For* ex vivo* coagulation assays, mice were treated with 20, 40, or 80 mg/kg body weight TMHE. An increase in APTT clotting time was observed with medium and high doses of TMHE 90 min after oral administration (Figure 3(a)). However, no significant changes were observed in PT clotting time (Figure 3(b)).

### 3.5. Clotting and Bleeding Times* In Vivo*

Compared to the control group, medium and high doses of TMHE significantly prolonged the clotting time, indicating that TMHE has anticoagulant effects (Figure 3(c)). Moreover, the dabigatran etexilate treated group had longer bleeding times than the TMHE treatment groups (Figure 3(d)).

### 3.6. Acute Oral Toxicity

No death was recorded in the 14 days observation period in the mice given 2 g/kg of TMHE orally. All of animals did not show any changes in the general appearance during the observation period.

## 4. Discussion

*Toona microcarpa* Harms has traditionally been used as an herbal medicine in Chinese culture for activating blood circulation to remove stasis. In this study, we used bleeding and clotting times in mouse models to investigate the* in vivo* hematological effect of TMHE. The results showed that TMHE significantly prolonged bleeding and clotting times in a dose-dependent manner, indicating that TMHE has potent antihemostatic effects. Hemostasis is divided into two consecutive stages: platelet aggregation and coagulation cascade. Therefore, both platelet and coagulation factors play roles in blood hemostasis. An increase in bleeding and clotting times suggests a defect or inhibition of either platelet aggregation or blood coagulation pathways. Firstly, we evaluated the potential antiplatelet activity of TMHE using ADP, collagen, and thrombin as agonists. The results showed that TMHE can inhibit platelet aggregation induced by thrombin, but not by ADP and collagen.

Secondly, the anticoagulant activities of TMHE were measured by APTT, PT, and TT. APTT is used to evaluate the coagulation factors such as VIII, IX, XI, XII, and prekallikrein in intrinsic coagulation pathway while PT is used to evaluate the coagulation factors V, VII, and X in extrinsic coagulation pathway [10]. TT reflects the blood coagulation status that transforms fibrinogen into fibrin, which is directly induced by the addition of thrombin. The test only detects disturbances in the final stages of coagulation, especially dysfibrinogenemia or the presence of thrombin inhibitors [11]. In our study, the results of APTT, PT, and TT assays* in vitro* showed that TMHE significantly prolonged APTT, PT, and TT clotting times in a dose-dependent manner; the* ex vivo* coagulation assays results showed that an increase in APTT clotting time was observed with medium and high doses of TMHE while no significant changes were observed in PT clotting time. These results indicated that TMHE may mainly exhibit anticoagulant activity correlating with the intrinsic coagulation process.

Thirdly, to further investigate the anticoagulant activity or mechanism of TMHE, coagulation factors (thrombin and FXa) inhibition tests were used. Thrombin and FXa are two highly validated targets that function at key steps in the coagulation cascade [12]. Many clinically used anticoagulant drugs are thrombin inhibitors (e.g., argatroban) or FXa inhibitors (e.g., rivaroxaban). Thrombin plays a central role in maintaining the integrity of hemostasis, interacts with most zymogens and their cofactors, and plays multiple procoagulant and anticoagulant roles in blood coagulation [13]. FXa, in combination with its cofactor Va, converts prothrombin to thrombin, resulting in initial fibrin formation. It sits at the junction of the extrinsic and the intrinsic pathways and plays a critical role in controlling the hemostatic network [4]. In this study, we found that TMHE inhibited thrombin activity in a dose-dependent manner but had no significant effect on FXa activity. Thrombin appears as the major target of the TMHE.

In summary, TMHE prolonged APTT, PT, and TT clotting times, inhibited the thrombin but not ADP or collagen stimulated platelet aggregation, and prolonged the whole bleeding and clotting time, possibly* via* inhibition of thrombin. These results will be helpful to understand the antithrombotic mechanism of* Toona microcarpa* Harms.

Many TCMs have been demonstrated to have anticoagulant activity. Danggui (*Radix angelicae Sinensis*), Honghua (*Flos carthami*), and Danshen (*Salvia miltiorrhiza Bunge*) are examples of TCM herbs that are used to activate blood circulation to remove blood stasis [14]. In addition, Taoren (*Persicae semen*) and Honghua (*Flos carthami*) used in pair named as Taoren-Honghua herb pair have also been used for many years to promote blood circulation to dissipate blood stasis [15]. In some studies, herbs extracts were found to have anticoagulant activities; for example, Xin et al. found that 95% ethanol extract of dragon's blood inhibits platelet aggregation and prolongs anticoagulation activities [16]. Zeng et al. found that the methanol extract of* Geum japonicum* at a concentration of 2 mg/mL has significant anticoagulant activity in the extrinsic coagulation pathway [17]. Han et al. found that 70% ethanol fraction from an aqueous extract of* Rubus chingii* leaves is the most antithrombotic fraction* in vitro* and* in vivo*, and flavonoids make an important contribution [18]. Wang et al. found that the* Erigeron breviscapus* extract has anticoagulant activity [19]. However, these studies mostly focused on their anticoagulant or antiplatelet activities, without implying the role of coagulation factors or platelet. Few studies further studied the effects of herb on the coagulation factors or platelet. Ku et al. found that persicarin and isorhamnetin which are isolated from* Oenanthe javanica* inhibit not only the activities of thrombin and FXa but also the generations of thrombin and FXa in human umbilical vein endothelial cells [20]. Robert et al. found that the leaf extracts especially the aqueous extract of* Croton zambesicus* Müell. Arg exhibited both the thrombin and FXa inhibition but no antiplatelet activity [21]. In this study, we also investigated TMHE's effect on coagulation factors or platelet and found that TMHE could inhibit the thrombin activity, but no effects on FXa or platelet.

In conclusion, this is the first report to demonstrate that TMHE has anticoagulant activity, most likely via its ability to inhibit thrombin activity.* Toona microcarpa* Harms may be a thrombin inhibitor that can function as an anticoagulant therapeutic. However, the anticoagulant activity of TMHE is weaker compared with positive control drugs such as heparin, argatroban, and dabigatran etexilate. It may be due to that TMHE is merely raw product which is extracted from the air-dried leaves of* Toona microcarpa* Harms by ethylacetate. We will perform further separation and purification for the active components.
